# Stature is inversely associated with self-reported diabetes in middle-aged Mexican women

**DOI:** 10.26633/RPSP.2017.32

**Published:** 2017-03-06

**Authors:** Karl P Puchner, Ruy Lopez-Ridaura, Eduardo Ortiz-Panozo, Isabel Vieitez, Martín Lajous

**Affiliations:** 1 Center for Population Health Research National Institute of Public Health CuernavacaMorelos Mexico Center for Population Health Research, National Institute of Public Health, Cuernavaca, Morelos, Mexico.

**Keywords:** Body height, diabetes mellitus, women, Mexico, Estatura, diabetes mellitus, mujeres, México

## Abstract

**Objective.:**

To investigate whether stature is associated with two highly prevalent cardiom- etabolic disorders—diabetes mellitus (DM) and high blood pressure (HBP) —in middle-aged Mexican women.

**Methods.:**

*We conducted a cross-sectional analysis of a sample of 93 481 middle-aged Mexican female teachers, all participating in the Mexican Teachers Cohort (MTC, or* ESMaestras*) study. We used a multivariable regression model to investigate the association of stature quintiles with the self-reported outcomes of DM and HBP.*

**Results.:**

After adjusting for birth cohort, ethnicity, family history, birthweight, occupation of household’s head during participant’s childhood, menopausal status, and geographical region of birthplace, stature was inversely associated with DM, with the odds for DM being 9% higher in the lowest stature quintile when compared to the highest stature quintile. Stratification for location of residence resulted in confirmation of the above-mentioned findings only in partici- pants living in urban environments.

**Conclusions.:**

We found an inverse association of stature with DM but not with HBP. Our data suggest that urban setting might be an important effect modifier of this association, which merits further investigation since it might provide valuable insights into the epidemiological transition occurring in developing countries.

## RESUMEN

***Objetivo*.:**

Investigar si la estatura está asociada a dos trastornos cardiometabólicos — diabetes sacarina e hipertensión arterial— muy prevalentes en las mujeres mexicanas de mediana edad.

***Métodos*.:**

Realizamos un análisis transversal de una muestra de 93 481 maestras mexicanas de mediana edad que participaban en el Estudio de la Salud de las Maestras (ESMaestras). Utilizamos un modelo de regresión multivariante para investigar la relación entre los quintiles de estatura y los resultados autonotificados de diabetes sacarina e hipertensión arterial.

Después de ajustar los datos en función de la cohorte de nacimiento, la etnicidad, los antecedentes familiares, el peso al nacer, la ocupación del jefe del hogar durante la niñez de la participante, el estado menopáusico y la zona geográfica del lugar de nacimiento, se observó una relación inversa entre la estatura y la diabetes sacarina, dado que las probabilidades de aparición de esta última fue un 9 % mayor en el quintil más bajo de estatura que en el quintil más alto. La estratificación en función de la residencia permitió confirmar los resultados mencionados solo respecto de aque- llas participantes que vivían en entornos urbanos.

**Conclusiones.:**

Encontramos una relación inversa entre la estatura y la diabetes saca- rina, pero no entre la estatura y la hipertensión arterial. Nuestros datos indican que el entorno urbano podría modificar considerablemente el efecto de esta relación, lo que amerita continuar con las investigaciones dado que podrían arrojar una luz valiosa sobre la transición epidemiológica en los países en desarrollo.

Various recent studies have found an inverse association between adult stature and such cardiometabolic diseases (CMDs) as high blood pressure (HBP) (1) and diabetes mellitus (DM) (2). These associations have been consistently observed in women, while findings in men remain inconclusive (3, 4). The underlying pathophysiology is still not completely understood. However, it has been suggested that, apart from genetic factors (5), unfavorable intrauterine and early life conditions might play a crucial role. In a series of papers, Barker has shown that intrauterine growth restriction and low birthweight (LBW) predispose, through fetal programming, to chronic disease in later life stages (6, 7). Additionally, it is well documented that individuals with LBW are more likely to exhibit short adult stature than individuals born with normal or high birthweight development of CMDs in midlife (9, 10). Though lowand middle-income countries bear the main burden of stunting and LBW and exhibit generally shorter national stature averages, little is known about the magnitude of these associations in the developing world, as most of the relevant studies have been conducted in industrialized countries.

Mexico faces an unprecedented epidemic of CMDs. In the last two decades the prevalence of hypertension in the general population almost doubled (from 23.8% to 43.2%), and the percentage of people suffering from DM increased from 6.7% to 14.4% (11). This phenomenon, occurring in the midst of a fast-paced epidemiological and nutritional transition, is likely to be mediated through drastic changes in lifestyle and diet, resulting in a steep increase of obesity and related cardiometabolic comorbidity (12). Nevertheless, as in other developing countries, this transition deviates from the classical Omran model (13), as the epidemic of CMDs in Mexico is found to coincide with still relatively high rates of LBWs, childhood deprivation, and stunting (14). This fact raises the question whether short stature that reflects intrauterine and/or childhood deprivation is contributing in the aforementioned context to the risk of developing DM and HBP in later life stages (15). Investigation of this question is of great relevance for public health policy, as identification of context-specific risk factors is crucial for improving prevention and effective control of CMDs (16).

Therefore, we investigated whether stature is associated with two highly prevalent cardiometabolic disorders— DM and HBP—in middle-aged Mexican women.

## MATERIALS AND METHODS

### Study population

The Mexican Teachers Cohort (MTC) (*Estudio de la Salud de las Maestras*, or *ESMaestras*) is an ongoing prospective cohort study of 115 315 female teachers 25 years and older in 12 states of Mexico that differ significantly from each other culturally and economically (17). The MTC began in 2 states in 2006 and expanded to 10 more states in 2008. Participants are members of an economic incentives program for public education teachers called the Teachers Incentives Program (TIP) (*Carrera Magisterial*). In 2008, 106 493 participants returned an extensive self-response questionnaire concerning sociodemographic and anthropometric characteristics, lifestyle, and medical conditions. Women with self-reported stature were eligible for our analysis *(n =* 95 394). In order to minimize implausible values and avoid extreme outliers, we restricted our analysis to the population between the 1st and 99th percentile of stature, excluding from the final analytic sample values either below or above these limits. The final analytic sample comprised 93 481 women.

### Assessment of stature

The study anthropometry is based on self-reports. Following the written instructions, the women were asked to report their stature (in cm). In a validation study (now undergoing review for possible publication), we had evaluated the validity of self-reported anthropometric parameters in a subset of 3 756 participants. The Pearson correlation coefficient between statures based on self-reports and standardized measurements made by technicians was 0.84. On average, women overreported their stature by 2.2 cm.

### Ascertainment of self-reported high blood pressure and diabetes mellitus

The participants were asked to respond to the following questions for the HBP outcome: “Have you ever had HBP diagnosed by a clinician?”; “If yes, did you receive treatment?”; and “Since when have you been diagnosed with HBP?” The same kind of questions were asked for the DM outcome. Self-reported DM and, to a slightly lesser degree, self-reported HBP are regarded as accurate information. Most of the validation studies have found very high specificity and moderate to high sensitivity for both self-reported diagnoses (18–20). In a random sample of 938 cohort members from Mexico City taking part in face-to-face interviews up to 18 months after responding to the 2008 questionnaire, concordance in reported HBP between questionnaire and interview was 91.6%. Furthermore, 3 099 confirmation questionnaires were sent to women from all 12 participating states who self-reported DM in the 2008 questionnaire. The confirmation rate for self-reported DM in the 2008 questionnaire was 89.2%.

### Assessment of covariates

On the questionnaire, the women recorded information that included their birthweight (low (< 2.5 kg), normal (2.5–4.0 kg), or high (> 4 kg); menopausal status; occupation of household’s head during their childhood (farmer/manual worker, public sector employee/teacher, private sector employee/other); family history of diabetes and HBP (“Did or do your mother, father, sister, brother, or children suffer from high blood pressure?”: yes/no; the same type of question was asked for diabetes/elevated glucose); and command of an indigenous language (“Do you or your parents speak an indigenous language?”: yes/no), which is a variable that is used in the Mexican context to define indigenous populations (21). In addition, the women reported the state born in, which was then assigned to the following geographical regions: Gulf, Southern Mexico, Central Mexico, Northern Mexico, or the Capital (Mexico City). The geographic classification legitimately reflects the country’s past and present regional imbalances in terms of socioeconomic development and cultural and dietary diversity. Information on date of birth (which was later classified into one of three birth cohorts: 1950s and before, 1960s, or 1970s and later) was obtained through the questionnaire and/or the TIP. These three decade-categories coincide with major socioeconomic and demographic changes in Mexican society and reasonably capture the age range of our study population. Information on the person’s living environment (urban/rural) was based on the participant’s current workplace location, which was obtained through the TIP.

### Statistical analysis

We categorized women into five stature quintiles: Q1, ≤ 152 cm; Q2, 153–156 cm; Q3, 157–159 cm; Q4, 160–163 cm; and Q5, > 163cm. For covariates with missing values in more than 1% of the study population, we created missing indicators, which were included in the regression models. This was done for the following covariates: birthweight (26.7% missing values); occupation of household’s head during childhood (23.0%); geographical region of birthplace (10.5%); family history of DM (15.8%); and family history of HBP (13.9%).

We calculated the distribution in percentages of categorical variables and median or mean with standard deviations (SDs) for numeric variables stratified by stature quintiles. We estimated odds ratios (ORs) and 95% confidence intervals (CIs) for DM and HBP from logistic regression models, with the highest quintile of stature being the reference category. In addition, quintile-specific ORs adjusted for multiple variables were estimated. Multivariable models were adjusted for the following seven potential confounders: birth cohort; command of an indigenous language; family history of HBP or DM; birthweight; occupation of household’s head during childhood; geographical region of the state where participants were born; and menopausal status.

We defined four models a priori to evaluate the relationship between stature and the two outcomes: Model 1 (birth cohort, command of an indigenous language, and family history of HBP or of DM); Model 2 (Model 1 + birthweight); Model 3 (Model 2 + occupation of household’s head during childhood + geographical region of the state where participants were born); and Model 4 (Model 3 + menopause). We conducted separate analyses for HBP and for DM.

The models selection was theorydriven, based on a directed acyclic graph. By incorporating the aforementioned seven covariates in our models, we were able to adjust for secular trends in stature; genetic, intrauterine, and early life sociodemographic factors; and menopausal status. All of these covariates are known to influence or be associated with both adult stature and chronic disease risk in midlife. We did not include any obesity marker in our adjustment model, as we considered higher susceptibility to obesity to be an intermediate on the causal pathway of the association of stature with CMDs. Controlling for an intermediate bears the risk of introducing bias by means of an unmeasured common cause of the intermediate and the outcome (22). In addition, analyses with Model 4 were stratified by living environment (urban/rural).

We used likelihood ratio tests to assess potential interaction between living environments and stature quintiles. The multivariable adjustment was repeated in a complete case analysis that excluded all participants with missing values for the covariates of control.

Finally, in order to reduce misclassification, we conducted a sensitivity analysis that restricted the outcome definition to only the individuals receiving medical treatment for the diabetes and HBP conditions. The level of significance was 1.5. All analyses were conducted using the SPSS Statistics 17.0 and Stata/IC 12 software packages.

All participants signed an informed consent form. The study was approved by the Research, Ethics, and Biosecurity Committee at the National Institute of Public Health. The MTC analytical datasets only contains de-identified data. The identity of participants cannot be established by the variables that are available for researchers collaborating with the MTC study.

## RESULTS

In our study population, the mean age was 42.7 years (SD, 7.7), and 7.7% of the participants said they had a command of an indigenous language, a percentage that is very close to the national average (23). Of the study population, 5.4% of them reported having DM, and the prevalence of HBP was 15.2%.

Taller women were younger and more likely to report normal or high birthweight. Command of an indigenous language was more frequent among shorter women, who were also more likely to be born in the Southern region of the country and to be the offspring of farmers and manual workers (Table 1).

**TABLE 1.** Characteristics (in number and percentage) of 93 481 participants in the Mexican Teachers Cohort study who answered the 2008 self-response questionnaire, stratified by stature quintile, Mexico

Variable	Q1^a^ ≤ 152 cm (*n =* 20 817)	Q2 153–156 cm (*n =* 22 321)	Q3 157–159 cm (*n =* 14 199)	Q4 160–163 cm (*n =* 19 122)	Q5 > 163 cm (*n =* 17 022)
Birth cohort					
1960s	10 936 (52.5)	11 611 (52.0)	7 111 (50.1)	10 119 (52.9)	8 946 (52.6)
1970s and later	5 196 (25.0)	6 175 (27.7)	4 129 (29.1)	5 954 (31.1)	5 491 (32.3)
Speaks indigenous language	3 324 (16.0)	1 705 (7.6)	762 (5.4)	860 (4.5)	567 (3.3)
Birthweight					
Low	1 492 (7.2)	1 117 (5.3)	637 (4.5)	840 (4.4)	651 (3.8)
High	217 (1.0)	360 (1.6)	310 (2.2)	540 (2.8)	752 (4.4)
Positive family history of					
High blood pressure	11 493 (55.2)	13 065 (58.5)	8 433 (59.4)	11 346 (59.3)	10 170 (59.8)
Diabetes	9 815 (47.2)	10 762 (48.2)	6 785 (47.8)	8 936 (46.7)	7 794 (45.8)
Occupation of household's head during participant’s childhood					
Manual (farmer/worker)	6 939 (33.3)	6 570 (29.4)	3 904 (27.5)	5 044 (26.4)	4 263 (25.0)
Geographic region of birthplace					
Southern Mexico	4 792 (23.0)	3 659 (16.4)	1 905 (13.4)	2 233 (11.7)	1 630 (9.6)
Central Mexico	4 783 (23.0)	5 882 (26.4)	3 740 (26.3)	5 106 (26.7)	4 373 (25.7)
Northern Mexico	1 410 (6.8)	2 874 (12.9)	2 300 (16.2)	4 064 (21.3)	4 780 (28.1)
Menopause	5 642 (27.1)	5 512 (24.7)	3 360 (23.7)	4 102 (21.5)	3 324 (19.5)

***Source:*** Produced by the authors from the study data.

a The minimum value of Q1 is 143 cm, and the maximum value of Q5 is 173 cm. The median of the five quintiles is, in order from Q1 to Q5: 150 cm, 155 cm, 158 cm, 161 cm, and 166 cm.

We found an inverse association of stature with DM. The prevalence of DM in the lowest stature quintile was 6.3%, while in the highest quintile the prevalence was 4.8%. HBP prevalence decreased from 16.2% in the lowest stature quintile to 14.4% in the highest stature quintile. In the unadjusted model, ORs for DM and HBP increased steadily and significantly with decreasing stature quintile. After adjusting for birth cohort, this trend continued to be statistically significant for both outcomes. Additional multivariable adjustment for genetic and intrauterine factors did weaken the association of diabetes with stature, but without changing results substantially. Finally, the inverse association of DM with stature was confirmed even after further adjustment for both socioeconomic and environmental factors and menopausal status, with the odds for diabetes remaining 9% higher in Q1 than in Q5. In contrast, expansion of the adjustment model by the aforementioned variables, particularly by occupation of household’s head during childhood and geographical region of birthplace, led to the disappearance of any significant trends in ORs in the case of HBP (Table 2).

**TABLE 2.** Logistic regression calculation of unadjusted and adjusted odds ratios and 95% confidence intervals for high blood pressure (HBP) and diabetes mellitus (DM) in participants in the Mexican Teachers Cohort study who answered the 2008 selfresponse questionnaire (*n* = 93 841), according to stature quintile (Q1–Q5), Mexico

	Q1	Q2	Q3	Q4	Q5	*P* for trend
HBP (%)	16.2%	15.8%	15.3%	14.1%	14.4%	
Unadjusted	1.15 (1.09–1.22)	1.12 (1.06–1.18)	1.07 (1.00–1.14)	0.98 (0.92–1.03)	Reference	< 0.001
Birth cohort adjusted	1.03 (0.97–1.09)	1.04 (0.98–1.10)	1.00 (0.94–1.07)	0.96 (0.90–1.02)	Reference	0.048
Model 1^a^	1.06 (1.00–1.13)	1.04 (0.98–1.10)	1.00 (0.93–1.06)	0.96 (0.90–1.02)	Reference	0.003
Model 2^b^	1.05 (0.99–1.12)	1.04 (0.98–1.10)	0.97 (0.93–1.06)	0.96 (0.90–1.02)	Reference	0.008
Model 3^c^	1.03 (0.96–1.10)	1.03 (0.97–1.09)	0.99 (0.93–1.06)	0.96 (0.90–1.02)	Reference	0.063
Model 4^d^	1.02 (0.96–1.16)	1.02 (0.96–1.08)	0.99 (0.92–1.05)	0.95 (0.80–1.01)	Reference	0.112
DM (%)	6.3%	5.7%	5.1%	4.7%	4.8%	
Unadjusted	1.33 (1.22–1.46)	1.19 (1.09–1.30)	1.06 (0.96–1.18)	0.98 (0.89–1.08)	Reference	< 0.001
Birth cohort adjusted	1.18 (1.07–1.29)	1.09 (1.00–1.21)	1.00 (0.91–1.10)	0.96 (0.87–1.06)	Reference	< 0.001
Model 1	1.12 (1.03–1.24)	1.05 (0.96–1.15)	0.95 (0.85–1.05)	0.94 (0.85–1.04)	Reference	0.001
Model 2	1.12 (1.03–1.23)	1.05 (0.95–1.16)	0.95 (0.86–1.07)	0.94 (0.86–1.05)	Reference	0.001
Model 3	1.11 (1.02–1.22)	1.05 (0.95–1.16)	0.96 (0.86–1.06)	0.95 (0.86–1.05)	Reference	0.002
Model 4	1.09 (1.01–1.21)	1.04 (0.94–1.14)	0.95 (0.85–1.06)	0.94 (0.85–1.03)	Reference	0.005

***Source:*** Produced by the authors from the study data.

a Model 1 = birth cohort + speaking an indigenous language + family history of high blood pressure or diabetes.

b Model 2 = Model 1 + birthweight.

c Model 3 = Model 2 + household's head occupation during childhood + geographical region of birthplace.

d Model 4 = Model 3 + menopause.

When stratifying by urban/rural environment, the prevalence of DM and HBP was higher in urban participants when compared with the corresponding stature quintile of the participants living in rural settings (Table 3).

The inverse association between stature and DM was solely observed in subjects living in an urban setting, with the rural/urban difference being significant (likelihood ratio test, *P* = 0.008). Stratification in the case of HBP yielded no strata-specific results, with the interaction test also being nonsignificant (*P* = 0.560).

Complete case analysis (that is, including only participants with no missing values for any of the covariates) yielded similar results, with individuals in the shortest stature quintile having 12% higher odds of reporting DM than did the reference group, after multivariable adjustment (OR = 1.12, 95% CI = 1.00–1.29, *P* for trend = 0.010). On the other hand, no significant results were observed in the case of HBP (data not shown). Finally, although the association of DM with stature quintiles experienced further attenuation when the definition of positive outcome cases was restricted to subjects who reported receiving treatment for the indicated disease, *P* for trend remained significant (*P* for trend = 0.026). The stricter outcome definition did not alter the results in the case of HBP, which again did not exhibit any significant association with stature quintiles (data not shown).

## DISCUSSION

In this large cross-sectional analysis of middle-aged Mexican women, we found an inverse association between adult stature and self-reported previously diagnosed DM. This association was evident among participants living in urban environments, but not in rural ones. With respect to our second outcome, HBP, our findings are not suggestive of any significant relation between it and stature, as all excessive risk in shorter women seemed to be explained by the controlling covariates. The prevalence of DM and HBP in our study population was relatively low when compared to the national averages (11), probably due to the relatively young age of our study participants and the self-report-based assessment method used.

Our results are in line with various other publications that suggest an inverse relation between adult stature and hyperglycemic disorders (2, 24). In particular, the limited evidence from developing countries clearly indicates an inverse association of short adult stature with metabolic disorders; however, with regard to HBP, the results seem to be rather inconsistent (25, 26).

**TABLE 3.** Multivariable logistic regression calculation of adjusted odds ratios and 95% confidence intervals for high blood pressure (HBP) and diabetes mellitus (DM) in participants in the Mexican Teachers Cohort study who answered the 2008 self-response questionnaire, according to stature quintiles (Q1–Q5), stratified by living environment (rural: *n* = 22 668; urban: *n* = 69 667), Mexico

	Q1	Q2	Q3	Q4	Q5	*P* for trend
Rural						
HBP (%)	14.1%	14.5%	13.3%	12.0%	14.1%	
Model 4^a^	0.93 (0.82–1.06)	0.95 (0.84–1.08)	0.88 (0.76–1.02)	0.81 (0.71–0.93)	Reference	0.894
DM (%)	5.8%	4.9%	4.9%	4.1%	4.6%	
Model 4	1.05 (0.86–1.29)	0.90 (0.73–1.10)	1.00 (0.80–1.27)	0.89 (0.71–1.11)	Reference	0.436
Urban						
HBP (%)	16.9%	16.3%	15.9%	14.6%	14.4%	
Model 4	1.03 (0.96–1.11)	1.04 (0.98–1.12)	1.02 (0.95–1.10)	0.99 (0.98–1.06)	Reference	0.141
DM (%)	6.5%	6.0%	5.1%	4.9%	4.8%	
Model 4	1.10 (0.99–1.23)	1.08 (0.98–1.21)	0.94 (0.84–1.06)	0.95 (0.85–1.07)	Reference	0.010

***Source:*** Produced by the authors from the study data.

a Model 4 = birth cohort + speaking an indigenous language + family history of high blood pressure or diabetes + birthweight + occupation of household's head during childhood + geographical region of birthplace + menopause.

Numerous pathophysiological mechanisms could account for the observed association. As stature can be understood as the biological result of a complex interaction of genetic factors and a multitude of intrauterine and childhood conditions, short stature might just be a proxy for other exposures. According to developmental theory, intrauterine and early life conditions that to a large extent determine adult stature could substantially influence the risk for DM in midlife, through metabolic programming (27). Metabolic programming is understood as an adaptive process that occurs in response to a nutritional stimulus/insult during a vulnerable period early in life. It is hypothesized that this adaptive process might cause an irreversible alteration of the regulation of certain hormonal systems (e.g., hypothalamic-pituitary-adrenal axis, leptin levels), thus leading to an increased predisposition to, especially, abdominal obesity and thus DM (28). Also, there is some notion from animal models that intrauterine growth restriction may be related to reduced beta-pancreatic cell mass and hence be predisposing, under diets rich in carbohydrates, to inadequate insulin secretion and the resulting insulin resistance (29).

Adjustment of the observed association for birthweight had only a minor effect on our results. This might suggest that other factors that induce nutritional stress during infancy but not in utero—such as famine exposure (30), chronic malnutrition, and recurrent gastrointestinal infections (31)—might have an independent effect. Nevertheless, decrease of the actual effect size of low birthweight as a result of erroneously recalled birthweights cannot be ruled out. Secular trends in height, on the other hand, seem to play an important role in the association under investigation, given that adjusting for birth cohort categories revealed a strong confounding effect. The observed association between stature and DM could also theoretically be due to differences in the genetic/ethnic background. However, our data fail to demonstrate a dominant role of genetic factors in explaining the association between stature and DM.

Controlling for command of an indigenous language and family history of DM, though leading to some attenuation, did not substantially change the results. Our questionnaire does not ask for specification of the first-degree family member (i.e., parents, siblings, or children) suffering from the outcome. Therefore, the categories of family history for which we controlled might have been too broad, making the adjustment for shared genetic pathways between DM and short stature imperfect.

Social and environmental factors of childhood had an important confounding effect on the association under investigation. This result highlights the independent impact that childhood socioeconomic and environmental conditions may have on the relation of stature with metabolic disease. Stratification for living environments revealed strata-specific effects. The prevalence of DM was higher in urban participants when compared with the corresponding stature quintile of the participants living in rural settings. While there was no significant relation between shorter stature and diabetes among individuals from rural areas, participants living in urban environments had a higher risk of reporting DM with decreasing stature.

Better access to health care services might provide an explanation for the higher DM prevalence generally found among the urban participants. However, that better access does not clarify why the significantly increasing trend of DM prevalence along decreasing stature quintiles is found solely in women living in urban settings. Similarly, in studies from other societies in epidemiological transition focusing on obesity, presence of an inverse association of stature with obesity was predominantly or exclusively found in metropolitan areas (15, 32). In this light, it could be hypothesized that rapid urbanization, resulting in drastic shifts in lifestyle and dietary patters during the life course, may crucially modify the effect of adult stature on DM risk.

Although we adjusted for a series of significant variables influencing both stature and chronic disease, there are important residual confounders not assessed in our study. Stressful emotional adversities during childhood (33), environmental factors such as air pollution (34), and pediatric disorders (35) are only some of potentially underlying causal factors of the observed association.

A further limitation of our study that warrants consideration is that we were not able to specify the association of stature components, meaning leg and trunk length, with the examined outcomes. Leg length is believed to be the component of stature that is most sensitive to early childhood environment, while trunk length constitutes a marker of pubertal environmental influences (36). Consideration of stature components in the analysis would help to better detect the potential causal insults for both shorter stature and increased cardiometabolic risk.

Similar to all large-scale epidemiological studies, most information used in this study is provided by the participant and is subject to error. The presence of the outcomes was ascertained through self-report and was not measured or verified by a clinician. Although validations of HBP and DM that were conducted in the cohort yielded a high concordance and high confirmation rate, respectively, the subsample characteristics and methods used diverged. In this light, accuracy of the outcome data might be impaired. However, multiple validation studies, conducted in different populations, suggest that accuracy of self-report for both conditions is reasonably high (18–20). Also, the higher level of education among MTC participants and the access to health services due to their insurance status make it possible to use self-administered questionnaires to collect high-quality data on clinical conditions.

In order to reduce the possibility of misclassification, we conducted a sensitivity analysis defining only women treated for DM or HBP as individuals with a positive outcome. This sensitivity analysis yielded results in line with our main analysis. By choosing a stricter outcome definition, we were able to partly correct for overreporting of disease. However, lack of awareness of disease and resulting underreporting remain important limitations of our study. Nevertheless, this misclassification is likely to be nondifferential, leading to an underestimation of the true effect of stature on DM risk. In addition, the mean self-reported stature of the participants included in the analysis was 157.5 cm (SD = 6.1), a figure 2.5 cm higher than the average obtained by measurement in our prior validation study. Though systematic overreporting cannot be excluded, this discrepancy might merely reflect the higher average age of the participant subsample in the validation study when compared with the entire study sample (37). However, even in the case of systematic overreporting, this is rather nondifferential with respect to the outcomes.

Finally, the cross-sectional study design, per se, does not allow final conclusions on causality and/or temporality. Though some evidence suggests a negative effect of chronic hyperglycemia on bone metabolism (38), the relatively young age of our population makes it rather unlikely that the observed association is a result of DM-related osteoporotic shrinkage.

Among the strengths of this study are undoubtedly its large size and the detailed information on relevant pre-exposure covariates. Also, in contrast to the vast majority of previously published studies, we were able to explore the modifying effect of the current living environment on the association under investigation. To our knowledge, it is the first large-scale investigation of the association of adult stature with two of the most prominent CMDs, DM and HBP, in Mexico.

### Conclusions

Our findings suggest an inverse association of adult stature with DM in middle-aged Mexican women who are currently living in urban settings. Verification of these results by clinical data obtained in the same study population is required in order to draw final conclusions on the validity of the observed association. Further research, in terms of prospective studies, on the significance of short stature as a context-specific risk factor for DM in different developing-country settings is needed in order to improve prevention and control of CMDs on a global scale. This additional evidence would also provide valuable insights into the complex epidemiological transition of developing nations and build knowledge on the long-term consequences of early life deprivation. Finally, future studies should explore the value of stature as a parameter in scoring models that predict the risk of DM, which would help physicians better identify high-risk patients.

#### Acknowledgments.

We are grateful first and foremost to all the Mexican Teachers Cohort participants for their time and commitment. We would also like to thank the Teachers Incentives Program (TIP) and particularly Director de Regulación Victor Sastré, as well as the 12 state TIP coordinators and all their staff, for their continuous support. We are also thankful to Andrea Luviano, Adriana Monge, and Martha Tamez for their support.

#### Funding.

None specifically for this project.

#### Disclaimer.

Authors hold sole responsibility for the views expressed in the manuscript, which may not necessarily reflect the opinion or policy of the *RPSP/PAJPH* or PAHO.
